# Bladder Acellular Matrix Prepared by a Self-Designed Perfusion System and Adipose-Derived Stem Cells to Promote Bladder Tissue Regeneration

**DOI:** 10.3389/fbioe.2022.794603

**Published:** 2022-06-22

**Authors:** Shuwei Xiao, Pengchao Wang, Jian Zhao, Zhengyun Ling, Ziyan An, Zhouyang Fu, Weijun Fu, Jin Zhou, Xu Zhang

**Affiliations:** ^1^ Department of Urology, The Third Medical Centre, Chinese PLA General Hospital, Beijing, China; ^2^ Medical School of Chinese PLA, Beijing, China; ^3^ Department of Urology, Hainan Hospital of Chinese PLA General Hospital, Sanya, China; ^4^ Beijing Institute of Basic Medical Sciences, Beijing, China

**Keywords:** bladder acellular matrix, perfusion system, adipose-derived stem cells, vascularization, tissue engineering

## Abstract

The bladder patch constructed with the bladder acellular matrix (BAM) and adipose-derived stem cells (ASCs) was incubated with the omentum for bladder reconstruction in a rat model of bladder augmentation cystoplasty. A self-designed perfusion system and five different decellularization protocols were used to prepare the BAM. Finally, an optimal protocol (group C) was screened out by comparing the cell nucleus residue, collagen structure preservation and biologically active components retention of the prepared BAM. ASCs-seeded (BAM-ASCs group) and unseeded BAM (BAM group) were incubated with the omentum for 7 days to promote neovascularization and then perform bladder reconstruction. Hematoxylin and eosin and Masson’s trichrome staining indicated that the bladder patches in the BAM-ASCs group could better regenerate the bladder wall structure compared to the BAM group. Moreover, immunofluorescence analyses demonstrated that the ASCs could promote the regeneration of smooth muscle, neurons and blood vessels, and the physiological function (maximal bladder capacity, max pressure prior to voiding and bladder compliance) restoration in the BAM-ASCs group. The results demonstrated that the self-designed perfusion system could quickly and efficiently prepare the whole bladder scaffold and confirmed that the prepared BAM could be used as the scaffold material for functional bladder tissue engineering applications.

## 1 Introduction

Congenital and acquired bladder diseases such as bladder exstrophy, neurogenic bladder, malignant tumors, and trauma may cause anatomical or functional damage to the bladder (Zhe et al., 2016; Shi et al., 2017). In severe cases, total cystectomy and urinary diversion are even required (Minervini et al., 2014; Adamowicz et al., 2019). While repair and reconstruction after cystectomy is a vast surgical challenge in the field of urology (Zhao et al., 2015; Hou et al., 2016). At present, enterocystoplasty is still the gold standard for bladder reconstruction, commonly associated with a series of complications, such as long-term bacteriuria, stone formation, fibrosis, electrolyte imbalance, urinary tract infection and malignancy (Zhu et al., 2010; El-Taji et al., 2015; Huang et al., 2016). The development of tissue engineering technology provides new opportunities for repair and reconstruction after bladder defects.

The core content of tissue engineering research includes the selection of scaffold materials and the cultivation and proliferation of seeding cells (Shokeir et al., 2010; Gentile et al., 2021). In recent years, the scaffold materials used in bladder tissue engineering research mainly include synthetic polymers and natural materials (Horst et al., 2017). The bladder acellular matrix (BAM) in natural materials removes the cellular components that caused immune rejection and retains the extracellular matrix (ECM) structure that is conducive to cell adhesion, growth, proliferation, and differentiation (Pokrywczynska et al., 2015). Therefore, the BAM is often used in bladder tissue engineering research. Mechanical, chemical, and enzymatic digestion treatments are commonly used to prepare BAM (Davis et al., 2018). Many studies have reported that mechanical delamination of bladder tissues with removing the muscular layer and serous membrane before bladder decellularization can make the decellularization processing more uncomplicated and straightforward. However, it also makes the prepared BAM structure incomplete (Camacho-Alonso et al., 2019; Pokrywczynska et al., 2019; Zhao et al., 2019). In the chemical and enzymatic digestion treatments, the elution effect of chemical and enzymatic reagents on cells is the critical process of decellularization. Various chemical and enzymatic reagents are used for decellularization, including Triton X-100, sodium dodecyl sulfate (SDS), sodium azide, hypotonic liquid, ammonium hydroxide, and RNAse/DNAse (Gilbert et al., 2006). The choice of decellularization reagents and decellularization time will affect the retention of the BAM’s collagen structure and biologically active components. Moreover, the invention of perfusion decellularization was a breakthrough in creating whole organ scaffolds, which contained many ECM components, including collagen, elastin, proteoglycans and glycoproteins (Rajab et al., 2020). However, the bladder acellular protocols are various, and the exploration and optimization of the decellularization protocol have never stopped.

In tissue engineering research, mature somatic cells and stem cells are the primary sources of seeding cells (Adamowicz et al., 2017). Autologous urothelial cells and smooth muscle cells are used as seed cells to construct tissue-engineered bladder among mature somatic cells. However, they often need to be invasively obtained, and the source is limited in some urinary system diseases (Pokrywczynska et al., 2018). Stem cells have a strong self-proliferation ability and multi-directional differentiation potential conducive to tissue repair and reconstruction. Therefore, stem cells are an ideal source of seed cells for tissue engineering (Smolar et al., 2018). Among various stem cells, adipose-derived stem cells (ASCs) are widely used in the research of bladder tissue engineering due to their comprehensive sources, easy harvest, and ability to differentiate into multiple cell lineages (Wang et al., 2019).

When the tissue-engineered graft constructed with scaffold material and seeding cells are transplanted into the body, its surrounding environment changes from the simple medium environment *in vitro* to the complex tissue fluid environment *in vivo*. The supply of nutrients and oxygen is the key to the survival of the grafts (Smolar et al., 2016). When the tissue thickness exceeds 0.8 mm, blood vessels are needed to transport nutrients and oxygen to ensure cell survival in the tissue (Song et al., 2014). Generally, the thickness of bladder grafts often exceeds 0.8 mm. After transplantation, the ischemia of grafts could cause fiber contraction, perforation, and urine leakage (Alberti, 2016). One promising approach was pre-vascularization of the constructed tissue-engineered bladder before transplantation (Novosel et al., 2011; Adamowicz et al., 2017). The peritoneal cavity might serve as a natural bioreactor to promote the vascularization of the scaffold and the formation of the fibrous membrane wrapped around the scaffold (Campbell et al., 2008; Gu et al., 2010; Zhe et al., 2016). At the same time, the ASCs could stimulate angiogenesis through differentiation into epithelial cells and paracrine activity (Cherubino et al., 2016; Hutchings et al., 2020). Early and rapid vascular network formation is crucial to maintain the graft’s vitality and long-term survival.

**SCHEME 1:**
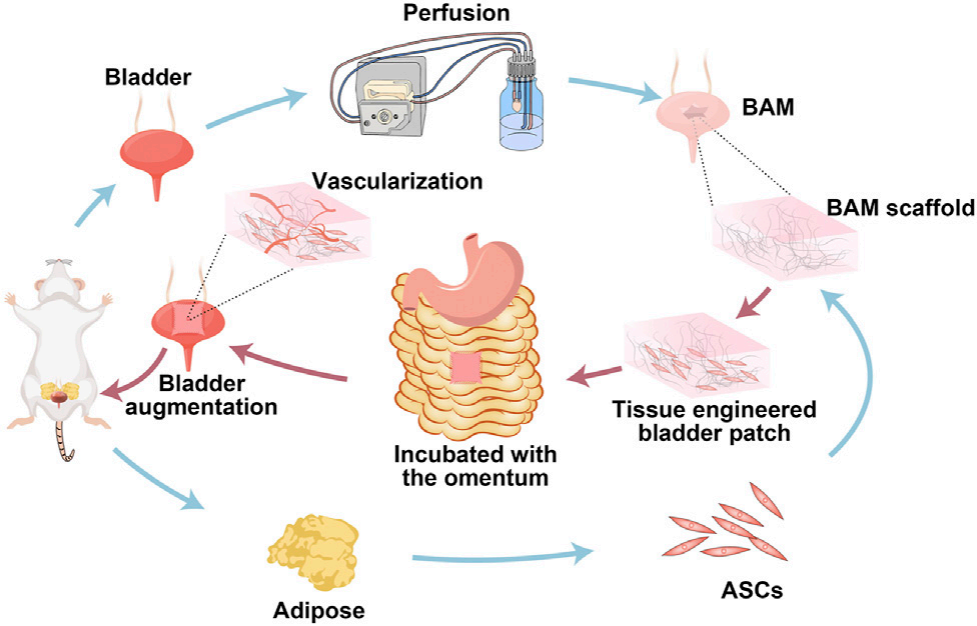
Schematic illustration on the preparation of the whole bladder scaffold by the self-designed perfusion system, and the tissue-engineered bladder patches incubated with the omentum for bladder augmentation.

In this study, we used a self-designed perfusion system and four different perfusion decellularization schemes to prepare the BAM. In addition, we also harvested the BAM with the commonly used decellularization method. The obtained BAM was evaluated in detail, including the cell nucleus residue, collagen structure preservation and biologically active components retention. Finally, an efficient perfusion decellularization protocol was selected. Moreover, the rat ASCs were isolated and cultured as seeding cells and planted on the prepared BAM to construct a tissue engineering bladder patch, which was incubated with the omentum to promote the neovascularization and then performed for augmentation cystoplasty to evaluate the repaired effect of tissue engineering bladder patch *in vivo* (Scheme 1).

## 2 Materials and Methods

### 2.1 Animals and Decellularization Processes

Animal experiments were approved by the Animal Experimental Ethics Committee of People’s Liberation Army General Hospital. The bladders were harvested from 7 to 10 weeks old Sprague Dawley (SD) rats and washed in phosphate-buffered saline (PBS). The fat and fibrous connective tissues of the outside surface and the urine in the bladder were wholly removed, and the bilateral ureters and urethral tissues connected to the bladder were reserved. Moreover, a perfusion system, mainly composed of a peristaltic pump, silicone tubes, 26-gauge needles and jar, was used to prepare BAM. Two 26-gauge needles were inserted into the bilateral ureteral lumens of the bladder, which was connected to the perfusion system to prepare BAM. The bladder samples connected to the perfusion system were then decellularized in four groups (group A-D). At the same time, the currently commonly used decellularized protocol was used to prepare BAM for comparative study (group E).

#### 2.1.1 Group A

The bladder samples were frozen at −80°C for at least 24 h, thawed at room temperature, and then connected to the perfusion system. Furthermore, the perfusion fluid flowed into the bladder through the bilateral ureters, filled the bladder, and then flowed out through the urethra in the perfusion system. During the decellularization process, the perfusion speed was controlled at 3 ml/min. The bladder samples were decellularized with 1% TritonX-100 (Sigma-Aldrich, St. Louis, MO, United States) for 6 h, and then with the deionized water for 1 h. Subsequently, 1% sodium dodecyl sulfate (SDS, Sigma) as perfusion fluid was used for 2 h. Finally, the bladder samples were rinsed with deionized water for 24 h to remove the detergent completely.

#### 2.1.2 Group B

The flow direction of the perfusate in group B was same as that in group A, while the difference was that the 1% Triton X-100 was used for 7 h and 1% SDS was used for 1 h. At last, the samples were rinsed with deionized water for 24 h to remove the detergent altogether.

#### 2.1.3 Group C

In group C, when the bladder samples were thawed at room temperature, the bladders were cut off the apex of the bladder and then connected to the perfusion system. The flow direction of the perfusate in the bladder was changed. The perfusate flowed into the bladder through the bilateral ureters and then flowed out directly through the channel on the apex of the bladder. The perfusate choice and the perfusion time were the same as those of group A. Finally, the detergents were thoroughly washed away.

#### 2.1.4 Group D

The bladder samples were also cut off the apex of the bladder, and the flow direction of the perfusion fluid in group D was the same as that in group C. In addition, the perfusate choice and the perfusion time were the same as those of group B. Finally, the detergents were thoroughly washed away.

#### 2.1.5 Group E

The commonly used decellularization protocol was used to prepare BAM (Li et al., 2008; Yuan et al., 2013; Leite et al., 2014). The bladder samples were frozen (−80°C) and thawed (37°C) three times in 5 mM ethylenediaminetetraacetic acid (EDTA, Gibco, Waltham, MA, United States) and 10 mM Tris HCl (Sigma). After that, the bladders were soaked in the 0.5% Triton X-100 and 26.5 mM ammonium hydroxide (Sigma) solution for 14 days, and the solution was changed every 3 days. Subsequently, the bladders were washed overnight in PBS with benzonase nuclease (Sigma) at 37°C. At last, the bladder samples were thoroughly washed with deionized water.

After the decellularization procedures, the prepared BAM was soaked in deionized water, sterilized with ^60^Co radiation, and stored at 4°C.

### 2.2 Evaluation of the Decellularization Efficacy

Native bladders and decellularized tissues were fixed in 10% formalin solution, dehydrated with graded ethanol, and embedded in paraffin. Slices sectioned into 4 μM were deparaffinized and used for histological examinations. Hematoxylin and eosin (H&E) and 4, 6-diamidino-2-phenylindole (DAPI) staining were used to evaluate the cellular nucleus residue. The collagen and glycosaminoglycan (GAG) distribution were observed by Masson’s trichrome (MTS) and Alcian blue staining. In immunofluorescence staining, slides were deparaffinized, blocked, and incubated with rabbit polyclonal antibodies against collagen Ⅰ, collagen Ⅲ, collagen Ⅳ, fibronectin, and laminin at 4°C overnight (antibodies information: Supplementary Table S1). Subsequently, the slides were incubated for 2 h at room temperature with fluorescein isothiocyanate (FITC)-conjugated secondary antibodies (mouse anti-rabbit IgG, Abcam, Cambridge, MA, United States). Photomicrographs were acquired by a Nikon Eclipse Ti2-U fluorescence microscope (Nikon, Tokyo, Japan).

#### 2.2.1 DNA Quantification

Native bladders (*n* = 4) and decellularized tissues (*n* = 4 for each group) were dabbed dry with tissue paper and weighted (Wu et al., 2011). According to the manufacturer’s instructions, the total genomic DNA in wet samples was extracted by a Genomic DNA Kit (Tiangen, China). DNA content was measured using a Nanodrop ND3300 spectrophotometer (Thermo Fisher Scientific, Waltham, MA, United States). The DNA quantity was express as ng/mg wet weight of the samples.

#### 2.2.2 Collagen Quantification

Native and decellularized samples (*n* = 4 for each condition) were weighed and transferred to the screw-capped tubes, which were incubated for 20 h at 95°C. The collagen content was quantified by a QuickZyme Total Collagen Assay Kit following the manufacturer’s instructions. Moreover, the absorbance was read at 570 nm on a microplate reader (Molecular Devices, Sunnyvale, CA, United States). Finally, the collagen content was express as μg/mg wet weight of the samples.

#### 2.2.3 Sulfated Glycosaminoglycan Quantification

Native and decellularized samples (*n* = 4 for each condition) were weighed and digested using a papain extraction reagent at 65°C overnight (Baiguera et al., 2014). The sulfated GAG was quantified by a Blyscan Sulfated Glycosaminoglycan Assay Kit according to the manufacturer’s instructions. In addition, the absorbance was measured at 656 nm on a microplate reader. Final values were expressed as μg/mg wet weight of the samples.

### 2.3 Scanning Electron Microscopy

The native bladders and decellularized matrix (Group C) were sectioned into small pieces and then fixed with 2.5% glutaraldehyde for 2 h at room temperature, washed twice with PBS, and dehydrated with gradient alcohol. The samples were freeze-dried and coated with 2 μM AuPd using a sputter coater system (sputter module 108auto, Cressington Scientific, Watford, United Kingdom). Images were captured with the scanning electron microscope (Nova200 Nanolab, China).

### 2.4 Adipose-Derived Stem Cells Isolation, Culture and Identification

The inguinal adipose tissues of three 2–3 weeks old SD were isolated and rinsed with PBS containing 100 U/mL penicillin (Sigma) and 100 μg/ml streptomycin (Sigma) three times. The tissues were minced and digested with 1 mg/ml collagenase type Ⅳ (Sigma) and dispase Ⅱ (Roche, Basel, Switzerland) at 37°C for 30 min with gentle agitation. The enzymatic activity was then neutralized by adding an equal volume of Minimum Essential Medium Alpha (α-MEM, Gibco) containing 10% fetal bovine serum (FBS, Gibco). Dissociated tissue was filtered to remove debris and centrifuged at 1000 rpm for 10 min. The collected cells were resuspended and planted onto the 10-cm culture dish in α-MEM with 10% FBS, and then incubated at 37°C with 5% humidified CO_2_. The culture medium was changed every 3 days. Third passage ASCs were used for flow cytometry identification. The ASCs were digested with 0.25% trypsin-0.02% EDTA, centrifuged at 1000 rpm for 5 min, and resuspended at a concentration of 106 cells/ml in PBS. Cells were incubated in the dark for 45 min at 4°C with the following primary antibodies: CD29, CD90, CD45, CD106. (antibodies information: Supplementary Table S1). After incubation, cells were washed three times with PBS and stored at 4°C before analysis using a FACSCalibur flow cytometer (Becton-Dickinson, United States). Data were analyzed using CellQuest software.

### 2.5 Cytotoxicity Test of the Prepared Bladder Acellular Matrix

The prepared BAM (Group C) was immersed in α-MEM at 37°C for 72 h, and the extract was collected for later use. The third passage ASCs were seeded in 96-well plates at a concentration of 5000 cells/well and incubated in α-MEM (containing 10% FBS) at 37°C with 5% humidified CO_2_ for 24 h. The culture medium was changed by BAM extract and control α-MEM respectively. At 1, 3, 5, 7 days, the culture medium was replaced by fresh α-MEM, ASCs were incubated with 10 μl of Cell Counting Kit-8 (CCK-8, Invitrogen, Carlsbad, CA, United States) reagent in 5% humidified CO_2_ for 2 h at 37°C. The absorbance at 450 nm was measured by a microplate reader. At the same time, live/dead staining was used to assess the ASCs viability in the BAM extract. At 3, 5, 7 days, the culture medium was aspirated, the ASCs were washed once with PBS and then incubated with 100 μl live/dead staining solution (Invitrogen) at 37°C with 5% humidified CO_2_ for 15 min, as recommended by the manufacturer. After washing with PBS, the ASCs were examined by Nikon Eclipse Ti2-U fluorescence microscope to distinguish the live and dead cells.

### 2.6 Labeling and Seeding of the Adipose-Derived Stem Cells

The ASCs were labeled with Cell Tracker CM-DiI (Invitrogen) to observe their distribution in BAM and trace the ASCs *in vivo*. The third passage ASCs were incubated with 2 mM CM-DiI for 5 min at 37°C and then for an additional 15 min at 4°C. After labeling, the cells were washed twice with PBS and resuspended with α-MEM containing 10% FBS for seeding. The freeze-dried BAM (Group C) was trimmed into 10 mm × 10 mm square pieces and placed in the 12-well plate. The labeled ASCs were seeded on the BAM pieces by dropping 30 μl of the cell suspension (1 × 10^6^ cells) onto the surface of the BAM piece, which was cultured at 37°C with 5% humidified CO_2_. After 4 h of co-cultivation, 2 ml of α-MEM containing 10% FBS were slowly added to the 12-well plate. The culture medium was changed every 2 days.

### 2.7 Distribution of Labeled Adipose-Derived Stem Cells on the Bladder Acellular Matrix Pieces

After 7 days of co-cultivation, the samples were washed twice with PBS and then scanned layer by layer with a confocal laser-scanning microscope (Nikon A1) to build the three-dimensional (3D) graphs and then observe the tridimensional distribution of ASCs inside the BAM piece. At the same time, the samples were fixed for 48 h in a 10% formalin solution, embedded in paraffin, and sectioned into 5 μM slices. The slices were stained with H&E and DAPI to assess the penetration depth of the ASCs in the BAM piece. Moreover, the samples were fixed with 2.5% glutaraldehyde, dehydrated with gradient alcohol, and then examined by SEM to observe the morphology of the ASCs on the surface of the BAM piece.

### 2.8 Animal Model

Thirty-six male SD rats (8 weeks old) were randomly divided into three groups: the BAM planted with labeled ASCs (BAM-ASCs group, *n* = 21), the BAM group (*n* = 9), and the cystotomy group (*n* = 6). The BAM-ASCs and BAM were sutured onto the omentum. The mucosal surface was faced toward the peritoneal cavity and the serosal surface toward the omentum. After 1 week, three rats from each group were sacrificed, and the incubated scaffolds were harvested and fixed in 10% neutral buffered formalin for histological examination. The other bladder patches incubated with omentum were used for bladder augmentation. The SD rats in the BAM-ASCs group were sacrificed at 2, 4, 12 weeks after transplantation (*n* = 6 for each time point). Meanwhile, Animals in the BAM and cystotomy groups were sacrificed at 12 weeks after implantation.

### 2.9 Bladder Augmentation

Briefly, the rats were anesthetized by an intraperitoneal injection of pentobarbital (30 mg/kg) and shaved to expose the surgical site. The surgical area was disinfected with iodophor and spread the sterile sheet. A 2 cm low abdominal midline incision was made, and the underlying tissue and peritoneum were dissected to expose the bladder. A 50% cystectomy was performed at the dome of the bladder to avoid damage to the trigone of the bladder. The bladder patches of BAM-ASCs and BAM incubated with omentum were used to repair this defect. The stitched area was marked by four non-absorbable 5–0 silk braided sutures (Johnson & Johnson, New Brunswick, NJ, United States) and then anastomosed by absorbable 6–0 sutures (Johnson & Johnson). In the cystotomy group, the bladder defect was anastomosed immediately after the incision. The watertight seal was confirmed by filling the repaired bladder with sterile saline *via* instillation through a 30-gauge hypodermic needle. Finally, the incision was sutured layer by layer.

### 2.10 Retrograde Cystography

The cystography was performed under general anesthesia by an intraperitoneal injection of pentobarbital (30 mg/kg). At the different time points, the bladder was emptied, and the contrast medium (30% iopamidol, Aladdin, China) was injected into the bladder by intravesical instillation until the stress incontinence. X-ray film was obtained from each bladder.

### 2.11 Cystometry Analysis

Bladder urodynamics was measured by conscious cystometry without anesthesia and restraint at 12 weeks post-implantation (Xiao et al., 2017). Briefly, the rats were anesthetized, and the bladder was extruded. One end of a PE-50 tube was inserted through the bladder dome and fixed by a purse-string suture, while the rest of the PE-50 was placed on the subcutaneous tunnel from the abdominal opening to the upper dorsum. The PE-50 tube was released and connected to an infusion pump and a pressure transducer through a three-way stopcock. The bladder was filled with the warm sterile saline by an infusion pump at a speed of 100 μl/min, and the intravesical pressure was recorded simultaneously. At least three micturition cycles were recorded. Urodynamic parameters including maximal bladder capacity (ΔV), max pressure prior to voiding (ΔP) and bladder compliance (ΔV/ΔP) were recorded and analyzed.

### 2.12 Histological and Immunofluorescence Analysis

At 2, 4, 12 weeks after implantation, the rats were euthanized, and the bladders were excised to prepared paraffin sections. Sections (4 μM) were stained with H&E and MTS. For immunofluorescence analysis, sections were deparaffinized, blocked, and incubated with rabbit polyclonal antibodies against cytokeratin AE1/AE3, α-smooth muscle actin (α-SMA), CD31 and NeuN at 4°C overnight (antibodies information: Supplementary Table S1). Afterward, the sections were incubated with species-matched FITC-conjugated secondary antibodies (Abcam), and the nuclei were counterstained with DAPI. Photomicrographs were acquired by a Nikon Eclipse Ti2-U fluorescence microscope. Photometric analyses, including the positive area of AE1/AE3, α-SMA, NeuN, and CD31-positive blood vessel density, were performed based on 6 randomly selected fields from 3 slides by Image J.

### 2.13 Statistical Analysis

Statistical analysis was performed using SPSS (17.0, United States). The results were expressed as mean ± standard deviation. The differences between groups were estimated using a two-tailed Student’s t-test or one-way analysis of variance with Bonferroni post hoc test. *p* values of less than 0.05 were considered statistically significant.

## 3 Results

### 3.1 Comparison of Decellularization Efficiency

**FIGURE 1:**
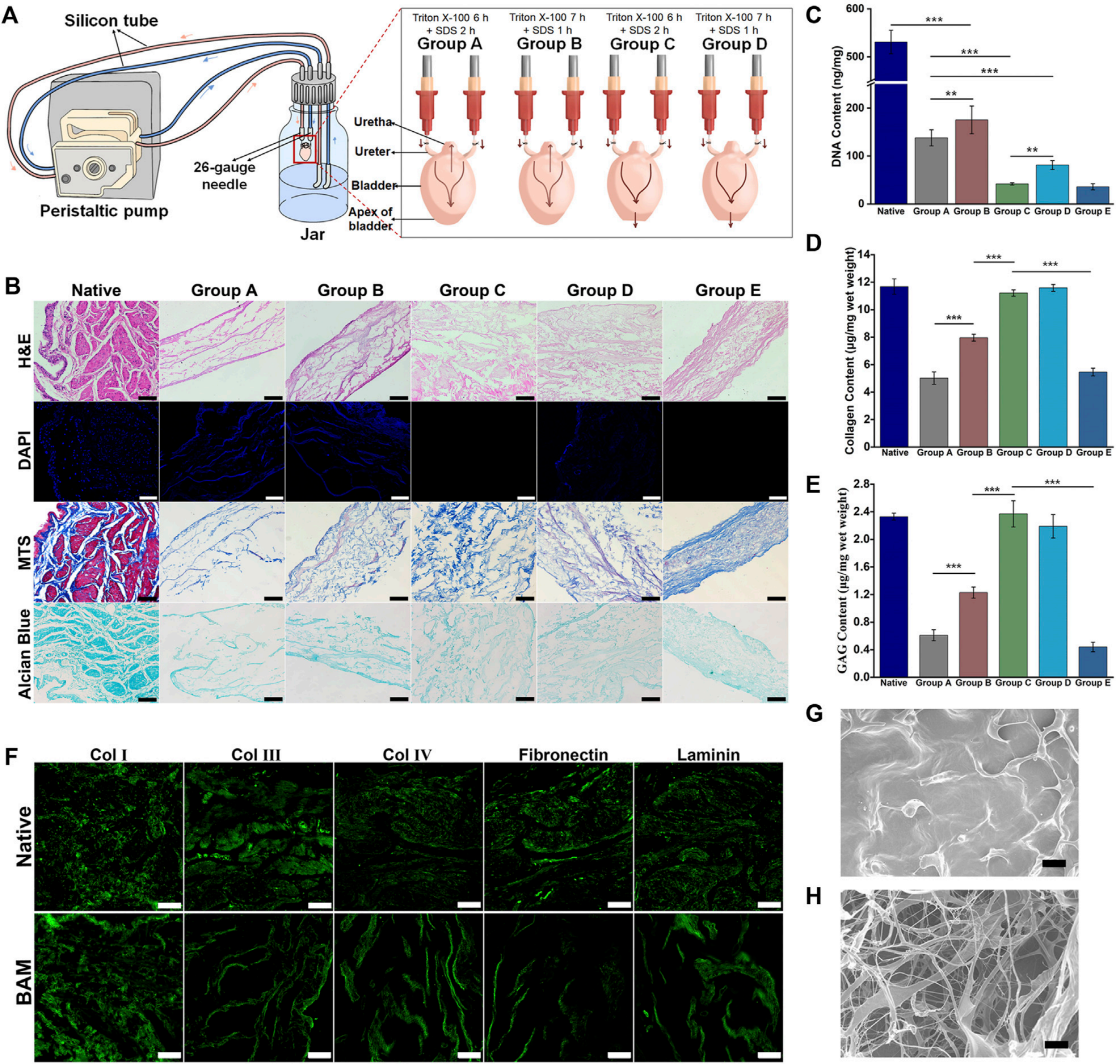
Evaluation of decellularization efficiency of the different decellularization groups. **(A)** The composition and connection of the perfusion decellularization system. The arrow indicated the flow direction of the perfusion fluid. **(B)** H&E, DAPI, MTS and Alcian Blue staining of the native bladder tissue and the BAM prepared by different acellular groups. The DNA content **(C)**, collagen content **(D)** and GAG content **(E)** of the native bladder tissue and the BAM prepared by different decellularization protocols. **(F)** Immunofluorescence staining of the BAM in group C with anti-collagen Ⅰ, anti-collage Ⅲ, anti-collagen Ⅳ, anti-fibronectin and anti-laminin. The SEM photomicrographs of the native bladder tissue **(G)** and the BAM **(H)** in group C. (B, F) Scale bar = 100 μM. (G, H) ×1000, scale bar = 10 μM. Significant difference is indicated as *p < 0.05, **p < 0.01, ***p < 0.001.

A perfusion decellularization system was designed and built to prepare the whole bladder scaffolds using the laboratory’s common equipment in this study (Figure 1A). The histological staining of the natural bladder and BAM prepared by different decellularization schemes was shown in Figure 1B. H&E staining indicated that the tissue structure of the BAM in groups A, B and E was significantly thinner than that of natural bladder tissue, while the thicknesses of the BAM in groups C and D were not different from that of the normal bladder tissue. DAPI staining suggested that the BAM in groups A, B and D had blue-stained nuclei-like structures. However, the BAM in groups C and E showed no residues of nuclei-like substances. MTS staining demonstrated red-stained muscle fiber structures and blue-stained collagenous fibers in the natural bladder tissue. The collagenous fibers could be observed in the BAM prepared by different groups without muscle fiber structures. Moreover, the collagen structure of BAM in groups C and D was significantly richer than that in groups A, B and E. In addition, Alcian blue staining confirmed the GAG retention in the BAM prepared by different groups.

The quantitative results of DNA confirmed that the DNA residues in the BAM prepared by different groups were lower than those in native bladder tissue (531.08 ± 24.59 ng/mg) (^***^ *p* < 0.001). Among the BAM in different groups, the DNA residues of BAM in group B (175.50 ± 28.89 ng/mg) were higher than those in group A (138.16 ± 16.83 ng/mg) (^**^ *p* < 0.01). In addition, the DNA residues were no significant difference between the BAM in group C (41.83 ± 2.25 ng/mg) and group E (35.91 ± 6.41 ng/mg). Moreover, the DNA residues of BAM in group D (81.25 ± 9.22 ng/mg) were higher than those in group E (^**^ *p* < 0.01) and lower than those in group A (Figure 1C). The quantitative results of collagen demonstrated that the collagen content of BAM in group C (11.21 ± 0.23 μg/mg) and group D (11.59 ± 0.25 μg/mg) was not significantly different from that of native bladder tissue (11.68 ± 0.56 μg/mg) and higher than that in groups E (5.46 ± 0.28 μg/mg) and B (7.96 ± 0.25 μg/mg) (^***^ *p* < 0.001). Moreover, the collagen content of BAM in group A (5.02 ± 0.45 μg/mg) was significantly lower than that in group B and no significantly different from that in group E (Figure 1D). The quantitative results of GAG showed that the GAG content of BAM in group C (2.37 ± 0.19 μg/mg) and group D (2.19 ± 0.17 μg/mg) was not significantly different from that of natural bladder tissue (2.33 ± 0.05 μg/mg) and higher than that in group B (1.23 ± 0.08 μg/mg) (^***^ *p* < 0.001). In addition, the GAG content of BAM in group B was higher than that in group A (0.61 ± 0.08 μg/mg) and group E (0.44 ± 0.07 μg/mg) (^***^ *p* < 0.001), while the GAG content of BAM in groups A was not significantly different from that in group E (Figure 1E).

Immunofluorescence staining indicated that the BAM in group C retained the typical ECM components, including collagen Ⅰ, collagen Ⅲ, collagen Ⅳ, fibronectin and laminin, among which collagen Ⅰ was the main component of the prepared BAM (Figure 1F). SEM showed that the surface of the native bladder was dense and relatively flat (Figure 1G). However, there was a random distribution of band-shaped fibers and a large number of pores with different sizes on the surface of BAM in group C (Figure 1H).

### 3.2 Cytotoxicity Analysis of Prepared Bladder Acellular Matrix to the Adipose-Derived Stem Cells

**FIGURE 2:**
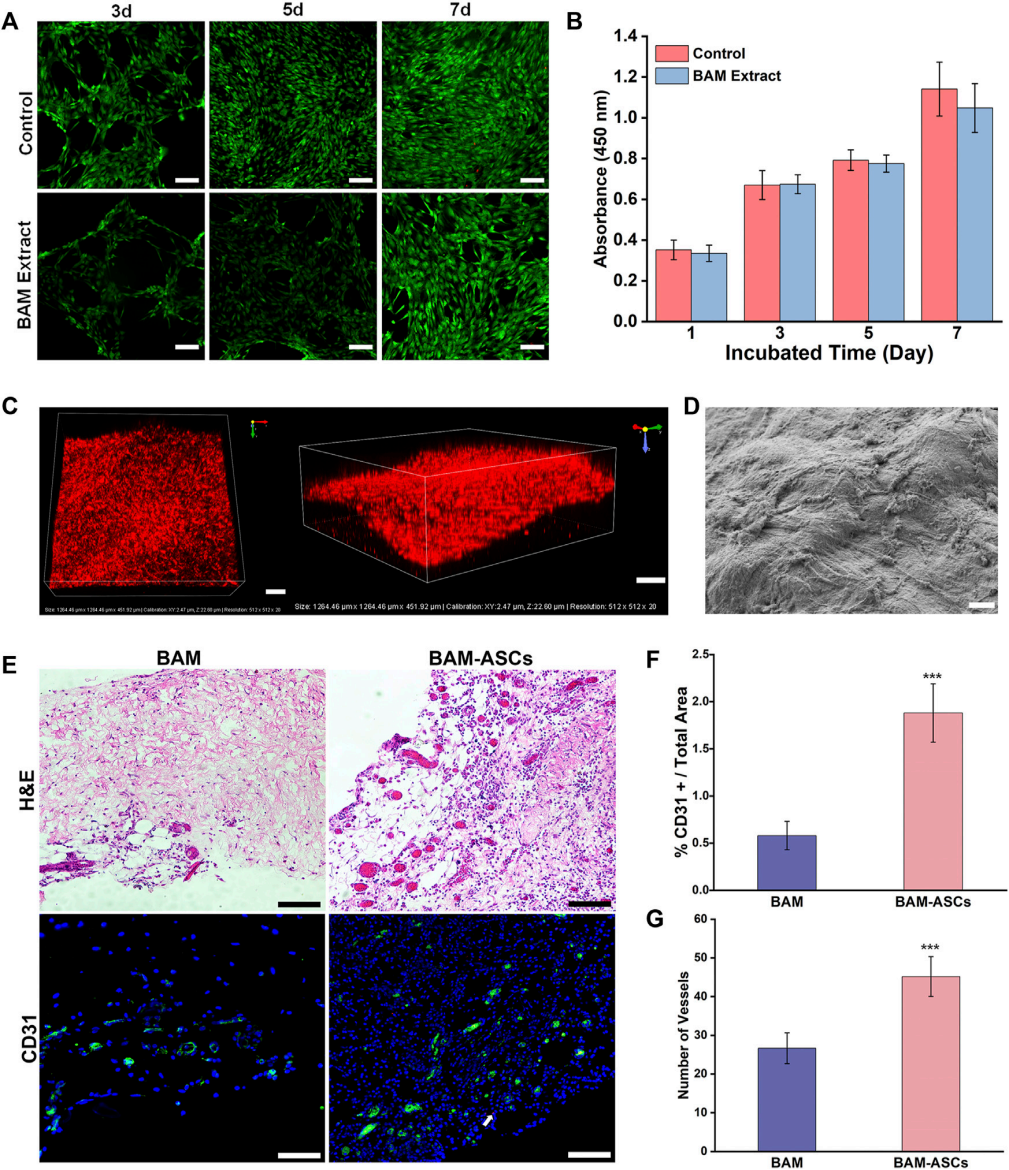
The growth and distribution of labeled ASCs in the BAM pieces and the vascularization of the constructed bladder patches incubated with omentum. **(A)** Live/dead detected the ASCs viability cultured in normal medium and the extract of BMA in group C. Green indicated the live cell. Red indicated the dead cell. **(B)** The cellular proliferative activity of the ASCs cultured in normal medium and the extract of BMA in group C. **(C)** The three-dimensional confocal photomicrographs of the labeled ASCs distributed on the BAM pieces. Red indicated the ASCs labeled with CM-DiI. **(D)** The SEM micrograph of the ASCs planted on the BAM pieces. **(E)** H&E and immunofluorescence staining of different bladder patches incubated with omentum for 7 days. The CD31-positive area was shown in green. White arrow: typical CM-DiI-labeled ASCs. The percentage of CD31-positive vessels area/total area **(F)** and the number of vessels **(G)** were compared among the different groups. (A, C) Scale bar = 200 μM. (D) ×1000, scale bar = 10 μM. (E) Scale bar = 100 μM. Significant difference is indicated as *p < 0.05, **p < 0.01, ***p < 0.001.

The isolated third-passage ASCs were uniform and showed a spindle-shaped morphology (Supplementary Figure S1A). The flow cytometry results confirmed that the isolated cells were positive expression for CD29 and CD90 and negative expression for CD45 and CD106 (Supplementary Figure S1B). Live/dead staining indicated that the ASCs cultured in the extract of BAM in group C had good cell viability without resulting in cell death at 3, 5, 7 days (Figure 2A). Furthermore, the CCK-8 test confirmed no significant difference in absorbance at 450 nm between the control and BAM extract groups at 1, 3, 5, 7 days (Figure 2B).

### 3.3 The Distribution and Growth of Labeled Adipose-Derived Stem Cells on the Bladder Acellular Matrix Pieces and the Vascularization of Bladder Patches Incubated With Omentum

H&E and DAPI staining confirmed that many labeled ASCs were distributed on the surface of the BAM pieces and formed into layers, and the labeled ASCs also could be observed on the deep side of the BAM pieces (Supplementary Figure S2). In addition, the 3D reconstruction graphs of the scaffolds demonstrated that the labeled ASCs were distributed on all layers of the scaffolds (Figure 2C). Furthermore, the SEM showed that many ASCs were tightly attached to the surface of the BAM pieces (Figure 2D). H&E and immunofluorescence staining could observe the formation of vascular structures in the BAM and BAM-ASCs groups, and the vascular structures in the BAM-ASCs group were more than those in the BAM group (Figure 2E). Moreover, the immunofluorescence analysis revealed that the percentage of CD31-positive vascular structures area/total area in the BAM group (0.58 ± 0.15%) was significantly lower than that in the BAM-ASCs group (1.88 ± 0.31%) (Figure 2F), and the number of CD31-positive vessels in BAM-ASCs group (45.17 ± 5.15) was more than that in BAM group (26.67 ± 3.98) (^***^ *p* < 0.001) (Figure 2G).

### 3.4 The Bladder Patches Incubated With Omentum Promoted Bladder Repair According to Gross Observation and Retrograde Cystography

**FIGURE 3:**
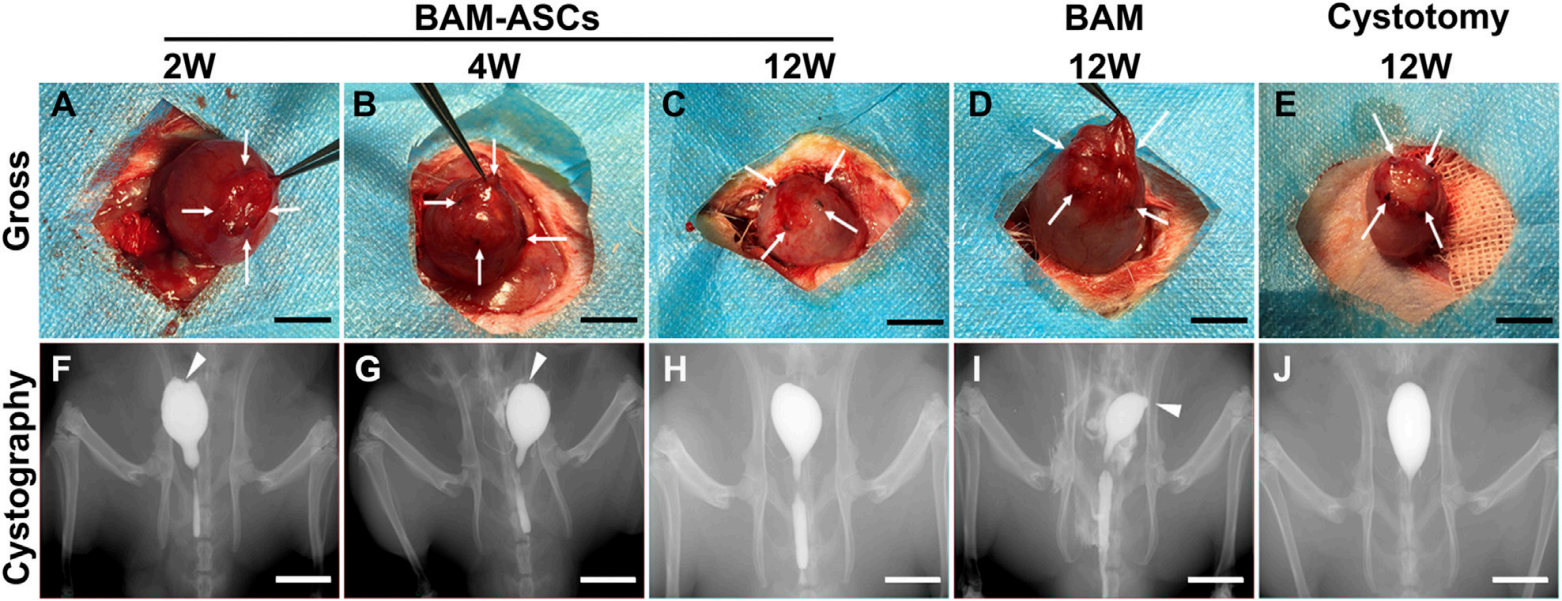
Different bladder patches prompted bladder morphology restoration. Gross photographs of the bladder repair area in the BAM-ASCs group at 2 **(A)**, 4 **(B)**, 12 **(C)** weeks and in the BAM **(D)** and cystotomy **(E)** groups at 12 weeks. The retrograde cystography photographs of the bladder tissue in the BAM-ASCs group at 2 **(F)**, 4 **(G)**, 12 **(H)** weeks and in the BAM **(I)** and cystotomy **(J)** groups at 12 weeks. White arrows: labeled the stitch boundary of the bladder repair area. White triangles: marked the notch and diverticulum on the cystography images. Scale bar = 1 cm.

When the bladder tissue was surgically exposed, trivial adhesions could be observed between the repaired area and the surrounding omentum and intestine. In the BAM-ASCs group, the patches were separated from the normal bladder tissue at 2 and 4 weeks (Figures 3A,B). In addition, the patches were merged with the normal bladder tissue, and the boundary was blurred in the BAM-ASCs group at 12 weeks, which was similar in the cystotomy group at 12 weeks (Figures 3C,E). However, the shape of the patches was irregular, and the boundary with the surrounding normal bladder tissue was evident in the BAM group at 12 weeks (Figure 3D). There was no sign of leakage examined by retrograde cystography in each group. The dome structure on the apex of the bladder was disappeared, and a clear notch could be observed at 2 and 4 weeks in the BAM-ASCs group (Figures 3F,G). The shape of the bladder was uniform, and the dome structure could be observed on the top of the bladder at 12 weeks in BAM-ASCs and cystotomy groups (Figures 3H,J). Nevertheless, an obvious diverticulum could be examined at 12 weeks in the BAM group (Figure 3I).

### 3.5 Histological Examination of the Bladder Patches Incubated With Omentum to Promote the Regeneration of the Bladder Wall

**FIGURE 4:**
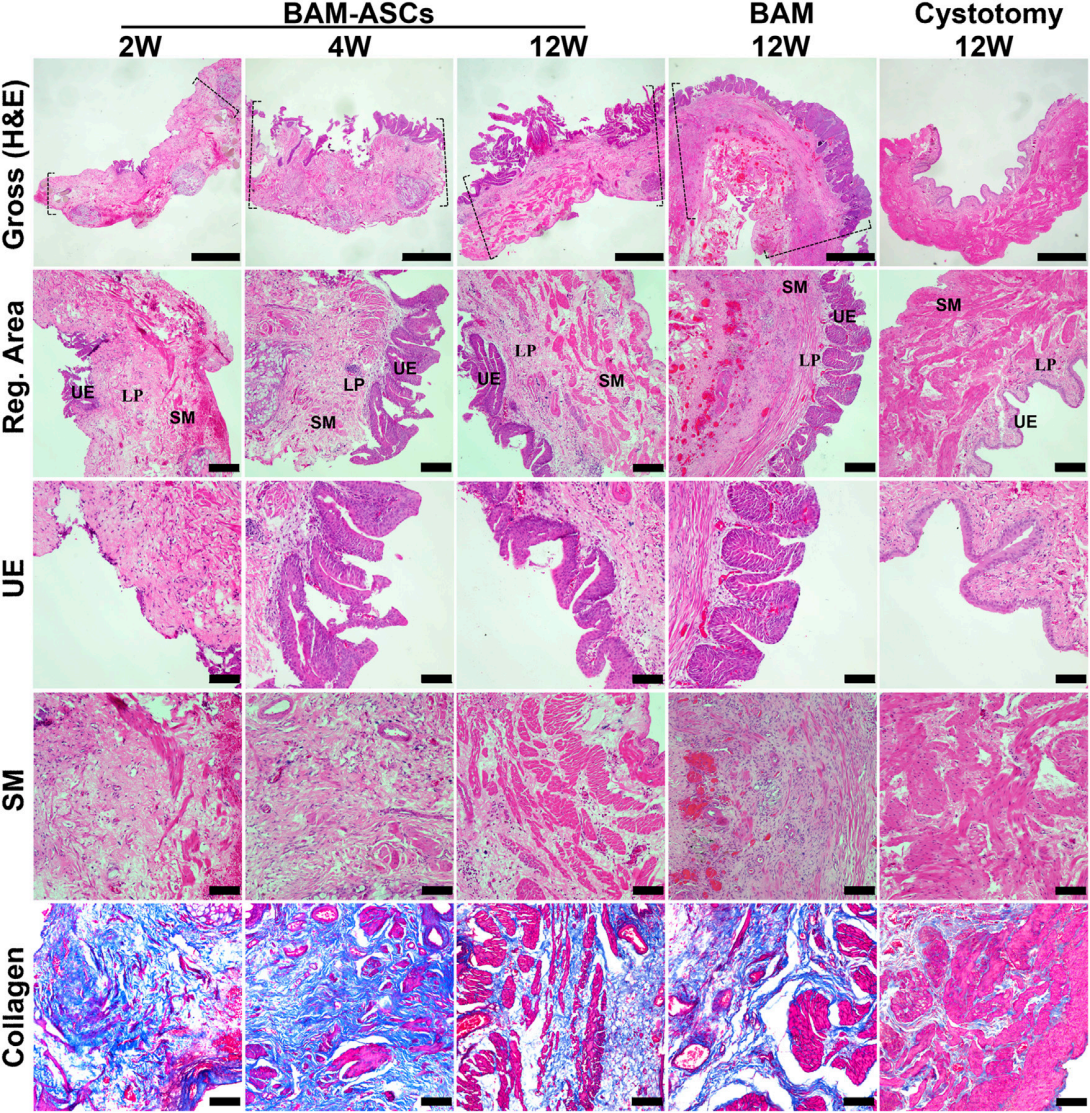
Histological evaluations of the regenerated bladder wall structure. Gross appearances of bladder tissue in the repair area and the regenerated areas of different groups (H&E). Histological staining of different structural layers of regenerated bladder wall in different groups, including urothelial layer (H&E), smooth muscle layer (H&E) and collagen tissue (blue in MTS). Dashed lines: margin of the repair area. UE: urothelial; LP: lamina propria; SM: smooth muscle. Gross photomicrographs: ×40, scale bar = 1 mm. Reg. area: ×100, scale bar = 200 μM. UE, SM and collagen: ×200, scale bar = 100 μM.

H&E and MTS staining showed fewer discontinuous urothelium cells, a large amount of fibrous connective tissue and a small amount of scattered smooth muscle tissue at 2 weeks in BAM-ASCs. At 4 weeks, a large number of proliferating urothelial cells and a small number of smooth muscle bundles could be observed in BAM-ASCs. In addition, there were apparent urothelial and muscular structures at 12 weeks in BAM-ASCs and cystotomy groups. Although the urothelial layer and muscular layer could be observed at 12 weeks in the BAM group, the urothelial cells were proliferated in the urothelial layer, and there were only a few smooth muscle bundles in the muscular layer (Figure 4).

### 3.6 Immunofluorescence Analyses of the Urothelium, Smooth Muscle, Nerves and Blood Vessels in the Regenerated Bladder Tissue

**FIGURE 5:**
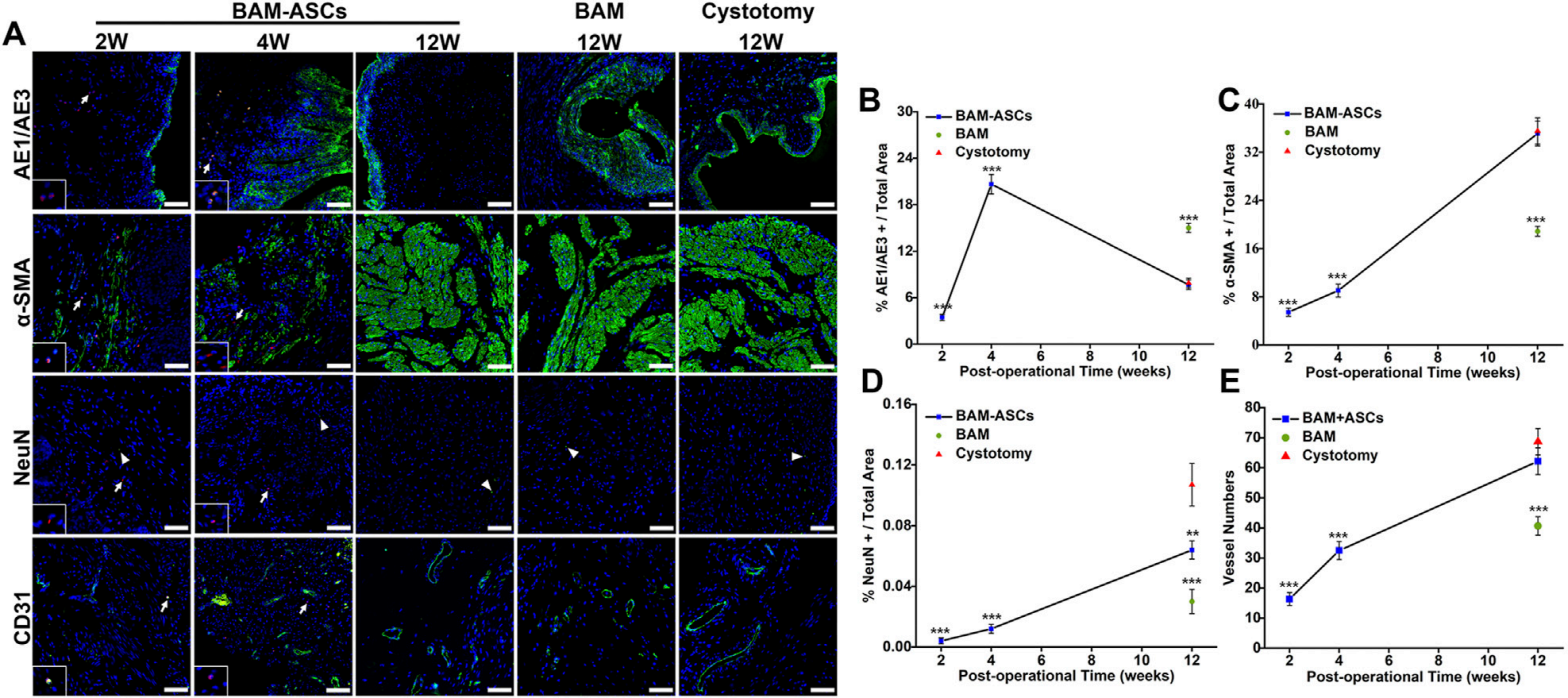
Immunofluorescence evaluations of the regenerated bladder tissue in the three groups. **(A)** Positive areas of AE1/AE3, α-SMA, NeuN and CD31 were showed in green (FITC), nuclei were indicated in blue (DAPI), and the ASCs were labeled in red (CM-DiI). Scale bar = 100 μM. The percentage per total field of AE1/AE3 **(B)**, α-SMA **(C)**, NeuN **(D)** and the vessel numbers **(E)** were quantitative analyses among the three groups. White arrows: CM-DiI-labeled ASCs. White triangles: showed the NeuN-positive neuron. Significant difference is indicated as *p < 0.05, **p < 0.01, ***p < 0.001 compared with the cystotomy group.

Immunofluorescence staining showed that the regeneration of bladder wall components in different groups (Figure 5A). CM-DiI labeled ASCs were not observed in the BAM-ASCs group at 12 weeks. The percentage of AE1/AE3 area/total area in the BAM-ASCs group at 4 weeks was higher than that at 2 and 12 weeks (20.64 ± 1.25% versus 3.45 ± 0.40% and 7.67 ± 0.61%, respectively, ^***^ *p* < 0.001). The percentage of AE1/AE3 area/total area in the BAM-ASCs group at 12 weeks was no different from that in the cystotomy group at 12 weeks (7.67 ± 0.61% versus 7.89 ± 0.63%, respectively) and lower than that in the BAM group at 12 weeks (7.67 ± 0.61% versus 15.00 ± 0.60%, respectively, ^***^ *p* < 0.001) (Figure 5B). In addition, the α-SMA-positive smooth muscle bundles in the BAM-ASCs group at 12 weeks were higher than that at 2 and 4 weeks (35.10 ± 2.05% versus 5.43 ± 0.68% and 9.03 ± 1.07%, respectively, ^***^ *p* < 0.001), and no significantly different from that in cystotomy group at 12 weeks (35.10 ± 2.05% versus 35.50 ± 2.18%, respectively). The α-SMA-positive smooth muscle bundles in the BAM group at 12 weeks were lower than that in BAM-ASCs and cystotomy groups at 12 weeks (18.84 ± 0.82% versus 35.10 ± 2.05% and 35.50 ± 2.18%, respectively, ^***^ *p* < 0.001) (Figure 5C). The number of NeuN-positive cells increased continuously with time (2, 4 and 12 weeks) after bladder reconstruction in BAM-ASCs group (0.004 ± 0.002%, 0.012 ± 0.003% and 0.064 ± 0.006%, respectively). The number of NeuN-positive cells in the cystotomy group at 12 weeks was higher than that in the BAM-ASCs group at 12 weeks (0.107 ± 0.014% versus 0.064 ± 0.006%, respectively, ^**^ *p* < 0.01) and significantly higher than that in the BAM group at 12 weeks (0.107 ± 0.014% versus 0.030 ± 0.008%, respectively) (Figure 5D). Furthermore, the number of CD31-positive blood vessels in the BAM-ASCs group at 12 weeks was higher than that at 2 and 4 weeks (62.17 ± 4.45 versus 16.33 ± 2.16 and 32.50 ± 3.02, respectively, ^***^ *p* < 0.001). The number of CD31-positive blood vessels in the cystotomy group at 12 weeks was no different from that in the BAM-ASCs group at 12 weeks (68.67 ± 4.41 versus 62.17 ± 4.45, respectively) and higher than that in the BAM group at 12 weeks (68.67 ± 4.41 versus 40.67 ± 3.08, respectively, ^***^ *p* < 0.001) (Figure 5E).

### 3.7 Bladder Capacity and Compliance Evaluation

**FIGURE 6:**
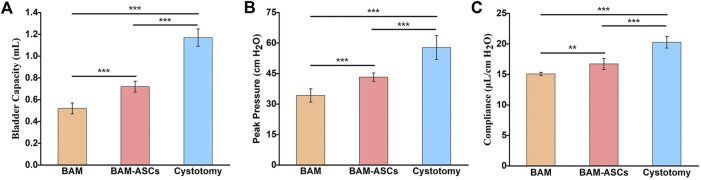
Long-term functional evaluations of bladder tissue repaired with different bladder patches. Bladder capacity **(A)**, peak pressure **(B)** and compliance **(C)** were tested at 12 weeks in the BAM-ASCs, BAM and cystotomy groups. Significant difference is indicated as *p < 0.05, **p < 0.01, ***p < 0.001.

The physiological function of the bladder in different groups at 12 weeks was examined through urodynamics. The bladder capacity in the BAM-ASCs group (0.72 ± 0.05 ml) was higher than that in the BAM group (0.52 ± 0.05 ml) (^***^ *p* < 0.001), while the bladder capacity in BAM-ASCs and BAM groups was lower than that in the cystotomy group (1.17 ± 0.08 ml) (Figure 6A). The peak pressure in the cystotomy group (57.81 ± 5.84 cm H_2_O) was higher than that in BAM-ASCs and BAM groups, while the peak pressure in the BAM group (34.23 ± 3.28 cm H_2_O) was significantly lower than that in the BAM-ASCs group (43.26 ± 2.02 cm H_2_O) (Figure 6B). The bladder compliance in the BAM-ASCs group (16.72 ± 0.91 μl/cm H_2_O) was higher than that in the BAM group (15.09 ± 0.25 μl/cm H_2_O) (^**^ *p* < 0.01), while the bladder compliance in BAM-ASCs and BAM groups was lower than that in cystotomy group (20.27 ± 0.95 μl/cm H_2_O) (Figure 6C).

## 4 Discussion

The ECM of different tissues and organs is both specific and similar. The specificity of the spatial structure and composition of the ECM determines the uniqueness of tissue functionality. However, the structure and composition of each specific ECM protein are highly conserved and similar among species, making it easy to identify within and between different species without causing the immune response (Gilbert, 2012). Therefore, the acellular ECM scaffolds have a wide of applications in the research of regenerative medicine. The decellularization for preparing ECM scaffolds included physical, chemical and enzymatic treatment methods (Guyette et al., 2014). Usually, several methods were combined to achieve a rapid and high-quality decellularization effect, which meant removing cellular components and preserving the structure and composition of ECM as completely as possible. Perfusion decellularization was widely used to prepare whole organ scaffolds, such as heart, liver, lung and kidney (Ott et al., 2008; Petersen et al., 2010; Uygun et al., 2010; Orlando et al., 2012). The principle of perfusion decellularization was to circulate the decellularization solution through the vascular network in the organs to achieve the acellular effect of the whole organ. However, the bladder tissue often lacked a suitable vascular network for perfusion, so the whole bladder scaffolds were difficult to prepare by perfusion decellularization. The SD Rats are the most commonly used experimental animals, and the bladder tissues of SD rat are suitable for exploratory research on perfusion decellularization. In addition, experimental animals often need to be sacrificed after the bladder tissue is removed, and it is more economical to use SD rats to carry out experiments than pigs or rabbits. In this study, the SD rats were chosen as a model.

A perfusion decellularization system was designed and built to prepare the whole bladder scaffolds using the laboratory’s common equipment in this study. The principle of this perfusion decellularization system was to simulate the normal physiological function of the bladder. The bilateral ureters served as the inflow tract, and the urethra served as the outflow tract, then the acellular fluid could flow through the entire bladder tissue to achieve the decellularization effect. Based on perfusion decellularization, physical and chemical methods were combined to screen out a fast and efficient decellularization protocol. The harvested intact bladder tissues were physically frozen and thawed to form intracellular ice crystals and then disrupt the cellular membranes (Gilbert et al., 2006). Nowadays, the commonly used chemical detergents for decellularization include non-ionic and ionic detergents. Triton X-100, the most widely used non-ionic detergent, has excellent infiltration ability but poor cleaning effect. SDS, one of the widely used ionic detergents, is very effective for removing cellular components from tissue, but long-time use may cause a decrease in the biologically active components and a loss of collagen integrity (Crapo et al., 2011). Therefore, two decellularization groups (group A and B) were used to evaluate the effect of different detergent types and washing time on the decellularization efficiency. However, histological staining and quantitative analyses in the two groups did not achieve the ideal decellularization effect. We hypothesized that when the bladder cavity was filled with the perfusion fluid during the circulatory perfusion decellularization process, the perfusion fluid pressure could stretch the collagen and further affect the collagen structure of the prepared BAM. In addition, when the deionized water was used to remove the chemical detergent, the detergent in the bladder cavity could not be quickly eliminated, so the nuclear materials eluted by the chemical detergent would deposit on the collagen skeleton. Therefore, the DAPI staining of the BAM in A and B groups showed blue-stained cell nucleus materials, and the DNA quantitative analysis further demonstrated that there was residual DNA in the BAM prepared by these two groups.

Based on the above analysis and the previous preparation experience of the ureteral acellular matrix (Xiao et al., 2016). We cut off the apex of the bladder to change the flow direction of the acellular fluid in the bladder, the bilateral ureters served as the inflow tract, and the apex of the bladder served as the outflow tract. The perfusate would not accumulate in the bladder cavity, and the pressure in the bladder cavity would decrease. Furthermore, we added two decellularization groups (group C and D) to evaluate the decellularization effect. Histological staining and quantitative analyses of DNA, collagen and GAG showed that the decellularization effect of group C was the most ideal compared with the other decellularization groups. Moreover, in group C, BAM retained the typical components of ECM, including collagen Ⅰ, collagen Ⅲ, collagen Ⅳ, fibronectin and laminin. In addition, the BAM in group C had no cytotoxicity, and the collagen structure had a large number of pores that facilitated the cell planting. Therefore, the BAM prepared by perfusion decellularized system in group C was selected to construct tissue engineering bladder.

The freeze-dried BAM in group C was used as the scaffold material, and the isolated ASCs were used as the seeding cells to construct tissue engineering bladder patches jointly. The internal structure of porous BAM after freeze-drying was similar to that of a dry sponge. When the cell suspension was dropped on the surface, the porous BAM facilitated the seeding cells into the interior. The histological staining and 3D reconstruction graphs confirmed that the labeled ASCs were aggregated on the scaffold’s surface and could penetrate the inside of the scaffold. However, when the constructed tissue-engineered bladder was transplanted into the body, serious complications may occur due to the lack of vascular networks, such as graft necrosis, fibrous contraction and perforation (Alberti, 2016). In this study, the constructed bladder patches were incubated with the omentum for 7 days to promote vascularization and fibrous membrane formation. The dense fibrous membrane could prevent urine leakage and protect the ASCs from the influence of the urine environment. Fluorescence staining and analysis confirmed that the vascularization effect of the BAM-ASCs group was significantly better than that of the BAM group. We considered that the ASCs and the omentum incubation played an essential role in promoting quick vascularization of the bladder patches.

The goal of tissue-engineered augmentation cystoplasty is to restore the physiological function of the bladder. Furthermore, the realization of the physiological function of the bladder is closely related to the structure of the bladder wall. The wall of the urinary bladder consists of four layers: the mucosa (urothelium), the submucosal connective tissue layer (lamina propria), the muscular layer and the serosal layer (Serrano-Aroca et al., 2018). The urothelium is a multilayered specialized epithelium, and the urothelial functions are a resilient and effective barrier against urine (Singh et al., 2018). Histological and immunofluorescence staining showed that the bladder patches in the BAM-ASCs group at 12 weeks formed a continuous urothelial layer, and the retrograde cystography confirmed that there was no sign of urine leakage. In addition, the immunofluorescence analysis demonstrated that the urothelial layer of the bladder patches in the BAM group at 12 weeks was significantly hyperplasia, while the urothelial layer of the bladder patches in BAM-ASCs and cystotomy groups at 12 weeks was no apparent hyperplasia. However, when the urothelial layer is injured, the natural urothelial cells can rapidly cover the damaged area (Smolar et al., 2016). Therefore, we hypothesized that the regenerated urothelial layer of bladder patches in each group resulted from natural urothelial regeneration. The ASCs may be involved in regulating the remodeling process of the urothelial layer by inhibiting the proliferation of urothelial cells.

The submucosal layer is composed of fibril-shaped or bundle-shaped collagens and contains a dense capillary plexus that maintains the vascular supply of the urothelium (Ajalloueian et al., 2018). Moreover, the muscular layer is the primary functional part of the bladder, and bundles of smooth muscle determine the bladder function during filling and micturition *via* their ability to relax and elongate (Smolar et al., 2016; Ajalloueian et al., 2018). In this study, histological staining and immunofluorescence analyses confirmed that the regeneration of smooth muscle, blood vessel structure and neurons in the BAM-ASCs group at 12 weeks was significantly more abundant than that in the BAM group at 12 weeks. The ASCs could promote the regeneration of smooth muscle, blood vessels and neurons, which was widely accepted in various studies (Kingham et al., 2007; Zhao et al., 2012; Chan et al., 2017). The physiological function of the bladder depends on the coordinated contraction and relaxation of the smooth muscle bundles and is regulated by the neuronal network (Xiao et al., 2017). Although the ASCs could promote the regeneration of neurons, the regeneration rate of the nerve in the BAM-ASCs group was unsatisfactory compared with the cystotomy group. Urodynamic evaluations are important tools for validating bladder function restoration and guiding scaffold optimization (Tu et al., 2012). In this study, urodynamic results confirmed that the capacity and compliance of the bladder repaired by BAM-ASCs at 12 weeks were significantly better than that by the BAM at 12 weeks. The results showed that the ASCs could increase bladder capacity and bladder compliance, which was consistent with the previous reports (Hou et al., 2016; Zhe et al., 2016). However, the bladder capacity and compliance in the BAM-ASCs group at 12 weeks were lower than that in the cystotomy group at 12 weeks. Due to the less nerve regeneration, the coordinated contraction and relaxation of regenerated smooth muscle bundles were bound to be affected, which could reduce the physiological function of the repaired bladder.

Omentum incubation and ASCs could promote the neovascularization of bladder patches, but the mechanism of ASCs in the bladder patches needed further study to elucidate. The bladder patches constructed by BAM and ASCs could regenerate the bladder wall structure, but the physiological function of the bladder repaired by BAM-ASCs was not very ideal. There are many types of neurons, and NeuN is only expressed in mature neurons and cannot fully reflect the neurons regeneration. Therefore, more markers could be selected to detect the neurons regeneration. We hypothesized that the few neurons in the bladder repaired by BAM-ASCs could not fully coordinate the relaxation and contraction of smooth muscle bundles. Therefore, the bladder patches constructed by BAM and ASCs should be further enhanced by neurotropic approaches. More research should focus on BAM’s rapid perfusion preparation in large animals to harvest the bladder patches with sufficient sizes or the tissue-engineered whole bladder and then substantiate the effect of bladder repair with long-term follow-ups to achieve the clinical application.

## 5 Conclusion

In this study, a self-designed perfusion system could prepare the whole BAM quickly and efficiently, and the BAM prepared by different groups was evaluated systematically and comprehensively. Finally, a suitable decellularization protocol was screened out. Moreover, the prepared BAM and the CM-DiI labeled ASCs were used to construct the tissue-engineered bladder patches, and the omentum incubation could achieve the vascularization of the constructed bladder patches. When these vascularized bladder patches were used for bladder augmentation, the ASCs planted on the bladder patches could promote the regeneration of smooth muscle, blood vessels and neurons. Furthermore, the vascularized bladder patches could promote morphological reconstruction, histological regeneration and physiological functional restoration. Consequently, the BAM prepared by self-designed perfusion system could be used as the stent material for the construction of bladder tissue engineering, and the constructed tissue engineering bladder patch was a promising scaffold and should be further optimized to achieve the clinical application.

## Data Availability

The raw data supporting the conclusions of this article will be made available by the authors, without undue reservation.
