# Supplementary material for: Bladder Acellular Matrix Prepared by a Self-Designed Perfusion System and Adipose-Derived Stem Cells to Promote Bladder Tissue Regeneration
Source: Front Bioeng Biotechnol. 2022 Jun 22;10:794603. doi: 10.3389/fbioe.2022.794603 (PMC9257038; doi:10.3389/fbioe.2022.794603)
Supplement: Supplementary file 1 [file DataSheet1.docx]

Supplementary Material

# 1. Supplementary Figures and Tables

## 1.1 Supplementary Tables

**Table S1.** Detailed antibodies information for flow cytometry and immunofluorescence analyses

| Against | catalog | Dilution |
| --- | --- | --- |
| CD29-FITC | 561796, BD Biosciences, Lake Franklin, NJ, USA | 1:200 |
| CD90-FITC | 561973, BD | 1:200 |
| CD45-FITC | 561867, BD | 1:200 |
| CD106-PE | 559229, BD | 1:200 |
| Collagen Ⅰ | ab254113, Abcam, Cambridge, MA, USA | 1:200 |
| Collagen Ⅲ | ab6310, Abcam | 1:200 |
| Collagen Ⅳ | ab236640, Abcam | 1:2000 |
| Fibronectin | ab268021, Abcam | 1:2000 |
| Laminin | ab11575, Abcam | 1:200 |
| AE1/AE3 | ab80826, Abcam | 1:200 |
| α-SMA | ab124964, Abcam | 1:1000 |
| NeuN | ab177487, Abcam | 1:3000 |
| CD31 | ab182981, Abcam | 1:2000 |

## 1.2 Supplementary Figures


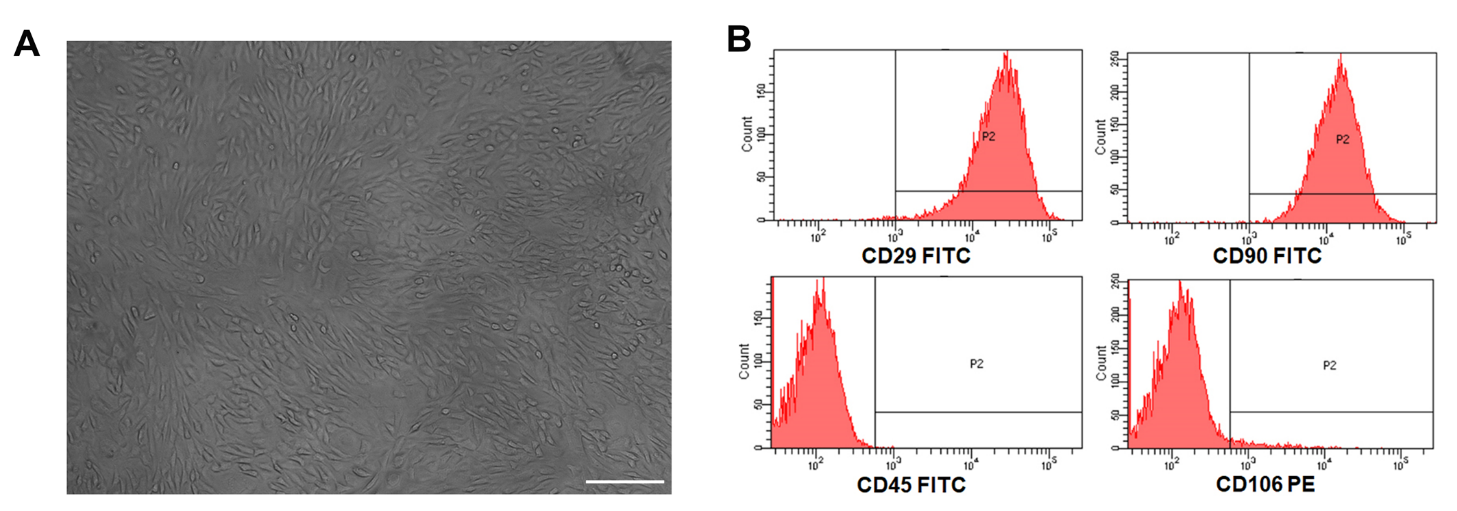


**Supplementary Figure 1.** Cultivation and identification of the ASCs. **(A)** The ASCs exhibited spindle-shaped morphology. **(B)** The isolated cells expressed CD29 and CD90, but negatively expressed CD45 and CD106. Scale bar = 200 μm.


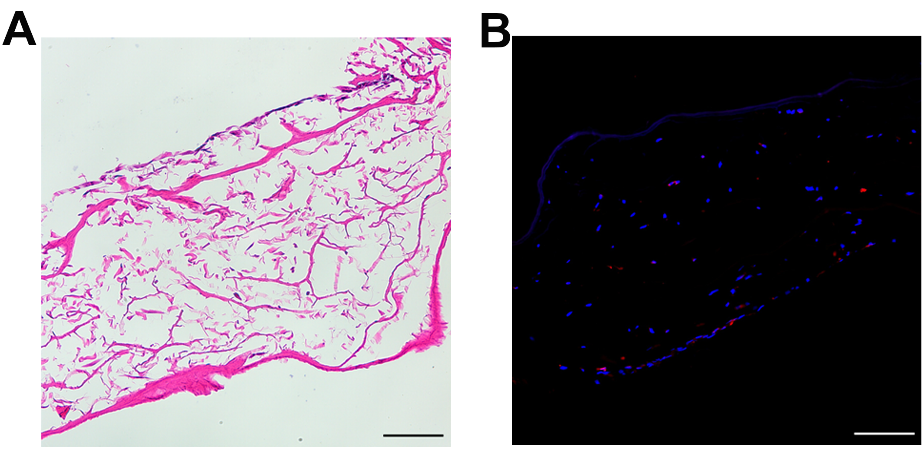


**Supplementary Figure 2.** Histological staining of the BAM planted with the labeled ASCs. H&E **(A)** and DAPI **(B)** staining of the BAM pieces co-cultured with the labeled ASCs for 7 days. Nuclei were stained in blue, and the red indicated the labeled ASCs in DAPI staining. Scale bar = 100 μm.
